# Editorial: Multi-omics and computational biology in horticultural plants: from genotype to phenotype, volume II

**DOI:** 10.3389/fpls.2024.1368909

**Published:** 2024-02-02

**Authors:** Yunpeng Cao, Xiaoxu Li, Hui Song, Muhammad Abdullah, Muhammad Aamir Manzoor

**Affiliations:** ^1^ School of Health and Nursing, Wuchang University of Technology, Wuhan, China; ^2^ Beijing Life Science Academy, Beijing, China; ^3^ Technology Center, China Tobacco Hunan Industrial Co., Ltd., Changsha, China; ^4^ Key Laboratory of National Forestry and Grassland Administration on Grassland Resources and Ecology in the Yellow River Delta, College of Grassland Science, Qingdao Agricultural University, Qingdao, China; ^5^ Queensland Alliance of Agriculture and Food Innovation, The University of Queensland, Brisbane, QLD, Australia; ^6^ Department of Plant Science, School of Agriculture and Biology, Shanghai Jiao Tong University, Shanghai, China

**Keywords:** multi-omics, computational biology, horticultural plants, genotype, phenotype

Horticulture, an integral component of the broader field of agriculture, has played a pivotal role in the development of human civilization. The shift from nomadic lifestyles to settled farming communities was greatly enabled by advancements in horticultural practices. This domain encompasses the scientific, technological, and artistic aspects of growing, breeding, processing, and commercializing various plant types, such as ornamental species, flowers, fruits, vegetables, nuts, seeds, and herbs. In recent years, there has been a surge in the sequencing of numerous horticultural plant genomes (Marks et al., 2021). The field of multi-omics and computational biology, particularly as they relate to horticultural plants and transition from genotype to phenotype, have experienced significant growth and diversification (Cao et al., 2022a). This progress has been driven by the incorporation of high-throughput technologies and innovative computational methods, yielding profound insights into plant physiological adaptation and biological mechanisms. The current Research Topic is focused on merging advanced omics and computational biology techniques to associate genotypes with phenotypes and link genetic markers to traits in various horticultural crops (**Figure 1**).

**Figure 1:**
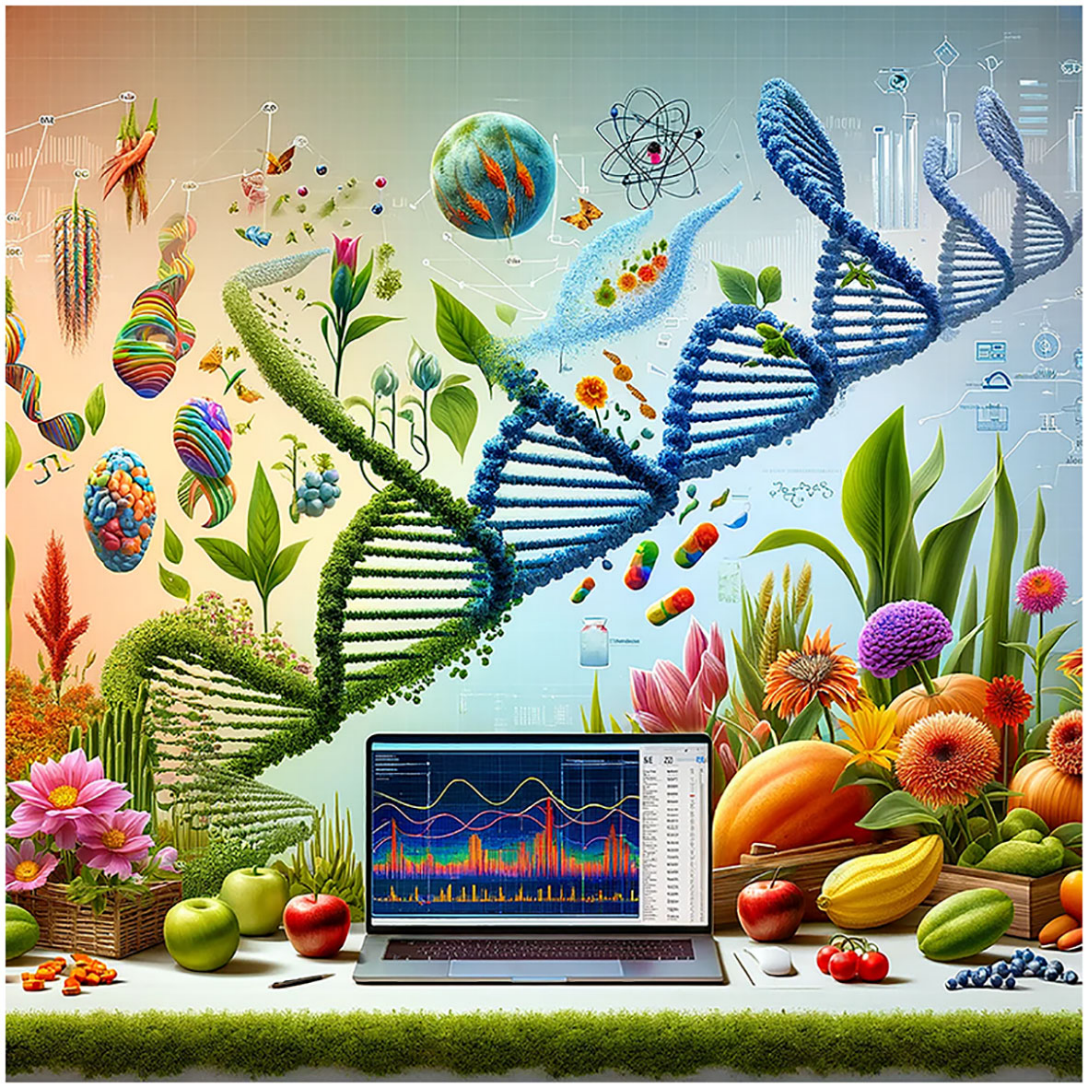
Multi-omics and computational biology in horticultural plants: From genotype to phenotype.

This Research Topic showcases a collection of 24 scholarly articles. Within this assemblage, two pieces are comprehensive reviews, while the other 22 constitute original research papers. Among these, a pair delves into the genome sequencing of horticultural crops. Three articles focus on the study of fruit crops, another trio sheds light on vegetable research, and a single paper explores the realm of Chinese herbal medicine. Additionally, one article is dedicated to ornamental crops, three examine various industrial crops, and the final nine provide insightful research on model crop species.

## Multi-omics data in horticulture plants

The integration of multi-omics data in horticulture significantly enhances our understanding of plant biology, leading to accelerated advancements in both research and breeding efforts within the field. This approach underscores the critical importance of combining various multi-omics datasets, such as genomics, transcriptomics, proteomics, and metabolomics, in horticultural plants. By combining various multi-omics datasets, offers a comprehensive view of complex biological processes, enhancing our understanding of plant growth, development, and environmental responses, and facilitates the identification of key genetic markers and traits for crop improvement, supporting innovative breeding strategies(Mondal et al., 2022). Moreover, it aids in more effective management of plant health and productivity, contributing to the overall advancement of horticultural science.

### Holistic insights

Integrating multiple omics approaches yields a holistic and comprehensive perspective of biological systems, enabling researchers to concurrently examine various biological layers, thereby deepening the understanding of plant biology. By analyzing genetic variations and their impact on gene expression, protein synthesis, and metabolite levels helps shed light on how these molecular changes translate into observable plant traits. This integrative analysis is crucial for unraveling the complex biological processes in plants, leading to significant advancements in fields such as plant biology, agriculture, and horticulture.

### Unraveling plant phenotypes

The integration of multi-omics data is crucial in revealing the intricate interactions and regulatory processes that shape plant phenotypes. For example, by correlating these omics data, researchers can discern the impacts of DNA sequence alterations on gene expression, protein synthesis, and metabolite concentrations (Cao et al., 2022a). Such insights shed light on how genetic variances contribute to the observable characteristics of plants. Yang et al. employed an Eigen Genome-Wide Association Study (EigenGWAS) methodology on a collection of 331 tomato varieties to uncover critical genetic determinants that affect metabolite variation throughout the tomato’s domestication and enhancement processes, thereby emphasizing the significance of polygenic selection in the developing of tomatoes with superior fruit quality (Yang et al., 2022).

### Elevating plant trait predictions

Enhancing the predictive capabilities of models that forecast plant traits can be significantly achieved through the integration of multi-omics data, and these models can more accurately account for the interactions among different biological processes. This comprehensive approach leads to more precise and accurate predictions of plant traits, acknowledging the intricate and interconnected nature of biological systems. For example, Jiang et al. elucidated the mechanism of polysaccharide biosynthesis of *Bletilla striata* by combining genomic and transcriptomic data (Jiang et al., 2022a). Furthermore, multi-omics analysis of potential R2R3-MYB transcription factors within the Euphorbiaceae family has successfully identified a specific MYB gene significantly involved in the biosynthesis of seed oil (Cao et al., 2023).

### Precision breeding advancements

Understanding the relationship between genotype and phenotype is fundamental for precise breeding design, as it lays the groundwork for predicting how genetic variations influence observable traits. The integration of multi-omics data significantly enhances precision breeding by identifying molecular markers linked to desirable traits in plants across various biological levels (Mahmood et al., 2022). This approach enables breeders to select traits with increased accuracy, thereby contributing to the development of superior plant varieties. By leveraging multi-omics data, breeders can gain a deeper understanding of the genetic basis of desired traits (Cao et al., 2022a). This holistic view allows for more informed selection processes and efficient breeding strategies, paving the way for the development of plants with optimized qualities.

### Unveiling plant-environment dynamics

The integration of multi-omics data enables researchers to gain a more comprehensive understanding of plant-environment interactions, revealing the effects of various environmental factors on gene expression, protein production, and metabolic processes. Such insights are crucial for understanding how plants grow, develop, and respond to stress. By analyzing these data, scientists can identify how external conditions such as temperature, moisture, and soil composition influence plant biology at multiple levels, which leads to a better understanding of plant adaptation and resilience (Großkinsky et al., 2018).

### Holistic insights for plant disease management

Integrating multi-omics data offers a holistic perspective on plant biology, significantly aiding in the diagnosis and management of plant diseases (Wang et al., 2021; Rathnasamy et al., 2023). This comprehensive approach can facilitate the identification of molecular markers associated with disease resistance, which are pivotal in guiding the development of disease-resistant plant varieties. At the same time, insights from multi-omics data also can inform and refine disease management strategies. By understanding the complex interactions at the genetic, transcriptomic, proteomic, and metabolomic levels, researchers can develop more effective methods to prevent and control plant diseases, leading to healthier crops and improved agricultural outcomes (Wang et al., 2021).

## From genotype to phenotype in horticulture plants

Integrating multi-omics data within horticultural studies offers a robust method for deepening our understanding of plant biology. In this context, we covered the crucial importance of multi-omics data integration in horticulture with a specific focus on tracing the journey from genotype to phenotype. This approach significantly enriches our comprehension of plant biology, ranging from the genetic foundation to observable characteristics, thereby enhancing the efficiency and depth of research and breeding programs in horticulture.

### Plant growth and development

To elucidate the genetic foundations of complex traits in plants, identifying candidate genes is a crucial step in modern genomic research. Previously, this task faced significant challenges due to the scarcity of high-quality genomic resources for various crops. However, the last twenty years have witnessed a revolutionary increase in the availability of detailed crop genomes and pan-genomes, which has greatly facilitated the process of connecting genotypes with phenotypes, a key aspect in understanding plant biology. Central to this understanding is the intricate network of gene interactions that orchestrate plant growth and development. Modern multi-omics techniques have become invaluable tools in deciphering this complexity.

Genomic studies, provide a comprehensive overview of a plant’s genetic makeup, facilitating the identification of key genes, such as those involved in the auxin signaling pathway. They establish links between genetic variations, including single nucleotide polymorphisms (SNPs) and insertions/deletions (INDELs), and observable traits (Zhang et al., 2014; Cao et al., 2022a). Complementary to genomic data, transcriptomics offers insights into gene expression patterns across various development stages of development and in response to environmental factors. Technologies such as RNA sequencing (RNA-seq) are instrumental in monitoring the expression dynamics of genes, including those associated with the auxin pathway, under different conditions (Cao et al., 2022b). Furthermore, metabolomics adds another layer to our understanding by analyzing the metabolic changes that occur during plant development and focusing on identifying active biochemical pathways and tracking the fluctuation of metabolites, including hormones like auxin. Together, these multi-omics approaches provide a holistic view of plant biology, linking genotype to phenotype and paving the way for advanced breeding strategies and crop improvement.

### Disease resistance pathways

In the realm of plant biology, a key area of study is the diverse and intricate pathways plants have evolved for disease resistance. This biological defense system is orchestrated through a complex network of genes, proteins, and metabolites, with each component playing a role in combating a wide array of pathogens. Multi-omics approaches are indispensable for dissecting and understanding these defense mechanisms at a molecular level.

Genomic studies in this area predominantly concentrate on *resistance* (*R*) genes and analyze genetic variations, such as SNPs and INDELs, to assess a plant’s innate disease resistance potential, as detailed by (Jiang et al., 2022b), with this genotypic analysis being essential for understanding the baseline resistance of different plant species or varieties. Complementing genomic data, transcriptomics provides a dynamic view of how plants respond to pathogen attacks at the gene expression level (Li et al., 2022). Utilizing RNA-seq, researchers track the expression patterns of R genes under pathogenic stress, which reveals the activation of the disease resistance pathway during an infection, offering insights into the temporal response of plants to pathogenic threats (Cao et al., 2021). Proteomics further enhances our understanding by examining the post-transcriptional changes in R proteins during pathogen attacks, elucidating the functional roles these proteins play in the plant’s defense mechanisms, and uncovering the biochemical processes involved in resisting pathogen invasion. Lastly, metabolomics completes the multi-omics picture by identifying the specific metabolites produced by plants in response to pathogen attacks, such as phytoalexins, thereby offering a comprehensive view of plants chemical defenses at the molecular level (Obata and Fernie, 2012). Collectively, these multi-omics approaches form a cohesive and detailed picture of plant disease resistance, providing vital insights for the development of more resilient crop varieties and innovative disease management strategies in agriculture.

### Stress response pathways

Plants, inherently stationary organisms, are subjected to a myriad of environmental stresses including drought, salinity, and extreme temperatures. The study of their molecular responses to such stresses is pivotal in advancing our understanding of plant resilience, particularly in the context of crop improvement. Multi-omics approaches offer an integrated and comprehensive set of tools for dissecting these complex stress response pathways.

At the genomic level, studies focus on identifying key genes that are activated or suppressed in response to environmental stresses, encompassing the discovery of diverse transcription factors that significantly influence the regulation of other stress-responsive gene expressions(Zhang et al., 2022). For example, brassinazole resistant 1 (BZR1) regulates the target gene *ethylene response factors 49* (*ERF49*) to enhance plant sensitivity to heat stress (Chen et al., 2022). Transcriptomics takes this analysis a step further by tracking the changes in gene expression under various stress conditions (Nye et al., 2023), which revealed how plants modulate their gene expression in real-time to adapt to and survive under adverse conditions. Proteomics adds another layer to our understanding of plant stress responses by examining proteins involved in these processes, including their abundance, modifications, interactions, and elucidating their regulatory roles and functional significance in the adaptation to stress (Kirk et al., 2022). Lastly, metabolomics complements these approaches by elucidating the metabolic alterations in plants under stress, pinpointing critical metabolites produced or modified in the plant’s defense, and serving as key indicators of stress response and adaptation (Obata and Fernie, 2012). Taken together, these multi-omics data offer a comprehensive perspective on plant responses to environmental stress, enhancing our understanding of stress tolerance mechanisms and facilitating the development of environmentally resilient crops.

## Future prospects of integrating multi-omics in horticulture plants

The integration of multi-omics into plant phenotyping marks the onset of a transformative epoch in horticultural research and practices, heralding a wealth of groundbreaking opportunities. For example, precision horticulture, increasingly practical through intricate multi-omics data, allows for the customization of each cultivation phase, from sowing to harvest, based on the unique genetic and environmental profiles of individual plants, thereby enhancing productivity, sustainability, and yield quality. Another is predictive breeding, where the amalgamation of multi-omics accelerates and refines the breeding process through predictive modeling that incorporates a wide array of genetic and phenotypic data. This methodology also significantly enhances the management of disease and stress responses in plants by establishing advanced early warning systems and pioneering new management strategies. Sustainable crop management, enabled by multi-omics, incorporates the complex interactions among plants, soil, and climate, fostering ecologically sustainable practices, reducing environmental impact, and enhancing plant biodiversity exploration, potentially uncovering new breeding and conservation resources. Collectively, the advancement of multi-omics democratizes access to sophisticated plant phenotyping techniques, thereby expanding the horizon of horticultural research globally and marking a significant advancement in optimizing horticultural practices and methodologies.

## Author contributions

YC: Validation, Writing – original draft, Writing – review & editing. XL: Writing – review & editing. HS: Writing – review & editing. MA: Writing – review & editing. MM: Writing – review & editing.
